# The Problem before the London Hospitals

**Published:** 1914-08-22

**Authors:** 


### The Problem before the London Hospitals.

The review of probable effects on the hospitals likely to be occasioned by the war, published in our columns last week, was scarcely in print before various announcements were made verifying the prophecies then made. Thus it quickly appeared that at several institutions practically the whole of the honorary staff were attached to the Territorial Force, whilst it was reported on good authority that at one hospital the entire resident staff had left for active service. At St. Bartholomew's the only chance of the institution being able to continue its work as under ordinary circumstances is that the Territorial hospitals to which its staff will be attached are located near by. More than any other, "Bart.'s" seems to be staffed with surgeons and physicians who became Territorial officers on the outbreak of hostilities. If, however, the military duties of the visiting staff are of such an engrossing nature, or centred in such a locality that they cannot keep in touch with their routine hospital work, it is likely that the consulting staff will resume active work at their *alma mater*, and so fill up the breach.

It so happens that one wing of this hospital is closed for cleaning, as usual at this time of the year, and the governors have informed the War Office that it is at their disposal from its reopening at the beginning of September. This block should provide accommodation for at least 200 patients. University College Hospital is also seriously handicapped owing to the number of men who have left, and further, owing to the fact that a large proportion of its visiting staff will have to take up duty with No. 3 base hospital of the Territorial Force whenever that should be constituted.

For the purposes of the Territorial Force London is divided into two parts, for each of which two large hospitals, each of not less than 500 beds, have to be provided. Under the present scheme one wing of the new King's College Hospital at Denmark Hill is a centre of one of these Territorial base hospitals. But obviously it cannot accommodate 500 wounded, and so provision has to be made either in the other parts of the hospital or in neighbouring buildings for the completion of the Territorial unit. St. Mary's Hospital has already provided a large number, probably a hundred men of all professional grades, who will serve as surgeons and dressers, a circumstance which will of course provide a great deal of work at that institution for those who are left behind. It is probable that some attempt to make up for the services of those men who have been taken from the hospitals will be made by utilising the services of volunteers in the shape of practitioners attached to the various medical schools who are offering part of their time daily to this purpose.

### With the Red Cross Expedition.

One of the most representative groups is the Red Cross detachment which, as we note elsewhere, went to Belgium under the direction of Sir Alfred Keogh, former Director-General of the Army Medical Department. This unit has been organised by the British Red Cross Society and consists of ten surgeons, ten dressers, and twenty nurses. Of the surgeons the London Hospital provided three, Messrs. Elliott, Taylor, and Austin; St. Thomas's sent Mr. Wyatt; whilst St. Bartholomew's sent no less than four—namely, Messrs. Attlee, Kemp, Neve, and Pascall; Edinburgh sending Messrs. Bloom and Erland. Sir Alfred Keogh should be able to get excellent work out of this fine band of workers, for some of those who have gone out as dressers went through the recent Balkan war. They will be assisted by nurses who have all had at least three years' experience, and who have been chosen by the London Hospital matron.

### Representative Names.

Among the many well-known men whose services have thus been called upon or who are likely to be engaged in military service at any time now may be mentioned Sir William Watson Cheyne, who is Lieutenant-Colonel of the 4th Territorial Hospital Corps for London, and who, it may be expected, will have to serve in connection with the hospital organised around King's College Hospital. The President of the Royal College of Surgeons, Sir Rickman Godlee, is Lieutenant-Colonel of the 3rd London Corps, in which he will have for his colleagues Sir Alfred Pearce Gould and Sir John Bland-Sutton, of the Middlesex, Sir John Ross Bradford, of University College, Sir Victor Horsley and Sir John Broadbent, of St. Mary's. Sir Bertrand Dawson, of the London Hospital, physician-in-ordinary to his Majesty, serves as Captain in the 2nd City of London Territorial Hospital Corps, having one of his colleagues at the "London," Sir Frederick Eve, as a senior officer. Another Middlesex representative, Sir J. Kingston Fowler, is Lieutenant-Colonel in the 3rd London Corps, while Sir William Arbuthnot Lane, of Guy's, is Captain in the 2nd City of London.

### Additional Help from London Hospitals.

Space precludes a fully detailed account of the various offers and distributions of beds made by the London hospitals; but it may be mentioned that amongst offers of help from this quarter not already mentioned the Great Northern Central Hospital at Holloway has set aside 100 beds for the needs of the War Office and Admiralty; whilst, should need arise, the London Homœopathic Hospital in Great Ormond Street is prepared to take in a considerable number of wounded soldiers and sailors. Further, the committee of the Metropolitan Ear, Nose, and Throat Hospital have also decided to maintain their accommodation in readiness for the reception of the wounded should their beds be required; and the Hospital for Epilepsy and Paralysis in Maida Vale is reserving fifty beds for a similar purpose. Last week some account of the preparations made at St. Thomas's Hospital was given in these columns, and such assistance as that institution was prepared to give must be considerably aided by the generosity of the two American ladies who have placed £5,000 at the disposal of the treasurer for the special assistance of the work in aid of those invalided home from military duties.

It is understood that Mr. Clayton Greene and Mr. V. Warren Low, of St. Mary's, who are devoting themselves to the care of the wounded, will take up work in Lord Tredegar's steam-yacht *Liberty*, which has been fitted up and accepted as a hospital ship at the expense of its owner, who has received a commission as a lieutenant in the Royal Naval Reserve. Practically all the hospitals have made offers of beds from the number of fifty and upwards; but in all cases where special reserves are being made for military contingencies committees of management and governors have been particularly anxious to make it known that proper provision has been made for the continued treatment of all accidents and urgent cases, such as are brought to them under ordinary circumstances.

